# Flower palate structure of the aquatic bladderworts *Utricularia bremii* Heer and *U. minor* L. from section *Utricularia* (Lentibulariaceae)

**DOI:** 10.1007/s00709-017-1097-9

**Published:** 2017-03-13

**Authors:** Bartosz J. Płachno, Małgorzata Stpiczyńska, Łukasz Krajewski, Piotr Świątek, Lubomír Adamec, Vitor Fernandes Oliveira Miranda

**Affiliations:** 1 Department of Plant Cytology and Embryology, Jagiellonian University in Kraków, 9 Gronostajowa St., 30-387 Kraków, Poland; 2 Faculty of Biology, Botanic Garden, University of Warsaw, Al. Ujazdowskie 4, 00-478 Warsaw, Poland; 3 Wetland Conservation Center, ul. Cieszkowskiego 1-3/31, 01-636 Warszawa, Poland; 4 Department of Animal Histology and Embryology, University of Silesia in Katowice, 9 Bankowa St., 40-007 Katowice, Poland; 5 Institute of Botany of the Czech Academy of Sciences, Section of Plant Ecology, Dukelská 135, CZ-37982 Třeboň, Czech Republic; 6 Faculdade de Ciências Agrárias e Veterinárias, Jaboticabal, Departamento de Biologia Aplicada à Agropecuária, Universidade Estadual Paulista (Unesp), São Paulo, Brazil

**Keywords:** Bladderwort, Carnivorous plant, Floral micro-morphology, Lentibulariaceae, Osmophore, Pollination, Sect. *Utricularia*, Ultrastructure

## Abstract

There is an enormous diversity in the structure of the flower palate of the carnivorous rootless genus *Utricularia*. This study aims to examine the structure of the palates in *Utricularia bremii* Heer and *U. minor* L of the *Utricularia* sect. *Utricularia*, which have a glandular palate type. In both species, the palate has only one type of glandular trichomes. Because of the occurrence of cell wall ingrowths in its glandular cells, any exudation may be transported via eccrinous secretion. It was proposed that the palate trichomes of the examined species act as scent glands and that the palate may play a role as an unguentarium. Both *U. bremii* and *U. minor* are of an open flower type. Thus, *U. bremii* and *U. minor* flowers can be penetrated by small, weak insects, which then easily have access to their generative structure. Small Hymenoptera (member of families Mymaridae and Braconidae) were observed as flower visitors of the male-sterile species *Utricularia bremii*.

## Introduction

A member of the genus *Utricularia*, subg. *Utricularia*, sect. *Utricularia* sensu Taylor (1989) is composed of the 34 species. However, two other species from this section have now been accepted as recognised species—*U. stygia* Thor and *U. tenuicaulis* Miki (Fleischmann 2012), and another new species was recently described—*Utricularia corneliana* R.W. Jobson (2012). In the case of their morphology, Taylor (1989) thought that the section *Utricularia* was derived compared to the other sections. Species from this section are suspended or affixed aquatic plants and that have a worldwide distribution. *Utricularia* species from section *Utricularia*, like other species from this genus, have a bilabiate corolla that extends posteriorly to form a floral spur. Most *Utricularia* have a palate that is the inflated base of the lower lip of the corolla and that differ both morphologically and sometimes also in terms of colour, from the remaining part of the perianth (Taylor, 1989; Płachno et al. 2016, 2017). Palates in the species from section *Utricularia* are very diverse morphologically, and five main types can be distinguished: a pubescent palate (e.g. *U. aurea*, *U. inflexa*, *U. stellaris*, *U. muelleri*, *U. geminiscapa*, *U. striata*, *U. floridana*, *U. poconensis*, *U. gibba*), a densely hairy palate (*U. reflexa*, *U. warmingii*), a glabrous palate (*U. raynalii*, *U. inflata*, *U. australis*, *U. intermedia*, *U. ochroleuca*), a glandular palate (*U. minor*, *U. bremii*) and an ill-defined palate (*U. biovularioides*, *U. cymbantha*, *U. naviculata*) (Taylor 1989). However, ultrastructural and histochemical data about the palates of the species from section *Utricularia* are sorely lacking. Recently, it has been shown that the prominent floral palate of *U. cornigera* and *U. nelumbifolia* (both from section *Iperua*) may function as an unguentarium—a structure that bears osmophores. However, palate could be also treated as a zone or area of osmophores in the flower when we choose nomenclature used by Endress (1994). In both of these species, the palate has a diverse micro-morphology that comprises unicellular, conical to villiform papillae and various types of multicellular, uniseriate, glandular trichomes (Płachno et al. 2017). Transmission electron microscopy further demonstrated that the palate papillae in these species may play a key role in providing the olfactory stimulus to attract insect pollinators due to the presence of relatively large, polymorphic plastids (chromoplasts) that contain many plastoglobuli. Moreover, the palate of some *Utricularia* members of sect. *Pleiochasia* may also function as an unguentarium, especially since it is papillose in these species. It also bears glandular trichomes in *U. uniflora* and *U. paulinae* (Płachno et al. 2016).

This study aims to examine the structure of the palate in two *Utricularia* species from the section *Utricularia*, which are the glandular palate type. In particular, it aims to ascertain whether these palates function as an unguentarium or they produce nectar to attract flower visitors. Another question is whether sterile male *U. bremii* invest in the glands to attract insects, because this species does not produce seeds. Thus, we chose the very closely related species fertile *U. minor* and sterile *U. bremii*.

## Material and methods

The species used in this study include *Utricularia bremii* Heer*—*collected from a shallow sand-pit Cep I in Suchdol nad Lužnicí, S Bohemia (Czech Republic), the Kuźnica Warężyńska sand-pit in Dąbrowa Górnicza (Poland) (Krajewski and Płachno, 2015); *U. minor* L.—collected from a fen bog in Nowa Wieś, Myszków (Poland). Plants with flowers were observed in natural condition in order to record flower visitors or pollinators. The insects were observed in the afternoon and photographed using a Canon PowerShot A480. Some additional material of *U. minor* from Herbarium KRA (voucher number 0401113) was also analysed.

### Floral structure and histochemistry

The distribution of the secretory glandular trichomes was determined by examining whole flowers using a Nikon SZ100 stereoscopic microscope.

Floral parts that bore glandular trichomes, namely the palate, were examined using light microscopy (LM), scanning electron microscopy (SEM) and transmission electron microscopy (TEM) as follows: Firstly, the epidermis of the floral palate was examined during anthesis, and pieces of the floral tissues were excised and fixed in 2.5% (*v*/v) glutaraldehyde/4% (*v*/v) formaldehyde in a 0.1 M sodium cacodylate buffer (pH 7.0) for several days, washed three times in a 0.1 M sodium cacodylate buffer pH 7 and post-fixed in a 1.5% (*w*/*v*) osmium tetroxide solution for 1.5 h at 0 °C. Dehydration using a graded ethanol series and infiltration and embedding using an epoxy embedding medium kit (Fluka) followed. Following polymerisation at 60 °C, sections were cut at 70 nm for transmission electron microscopy (TEM) using a Leica ultracut UCT ultramicrotome, stained with uranyl acetate and lead citrate (Reynolds 1963) and were examined using a Hitachi H500 transmission electron microscope at an accelerating voltage of 75 kV.

Semi-thin sections (0.9–1.0 μm thick) were prepared for light microscopy (LM) and stained for general histology using aqueous methylene blue/azure II (MB/AII) for 1–2 min (Humphrey and Pittman 1974) and were examined with an Olympus BX60 light microscope. The periodic acid-Schiff (PAS) reaction was also used to reveal the presence of insoluble polysaccharides, and Sudan Black B was used to detect the presence of lipids (Jensen 1962). Staining for total proteins was performed using Coomassie brilliant blue R250 or Ponceau 2R (Fisher 1968; Ruzin 1999).

Nikon Eclipse Ni-U and Olympus BX60 microscopes were used for the general photography and micrometry/photomicrography, respectively.

For SEM, the representative floral parts were fixed (as above or in 70% ethanol) and later dehydrated and subjected to critical-point drying using liquid CO_2_. They were then sputter-coated with gold and examined at an accelerating voltage of 20 kV using a Hitachi S-4700 scanning electron microscope (Hitachi, Tokyo, Japan), which is housed in the Scanning Microscopy Laboratory of the Department of Biological and Geological Sciences, Jagiellonian University in Kraków).

## Results

### *Utricularia bremii*

**Fig. 1:**
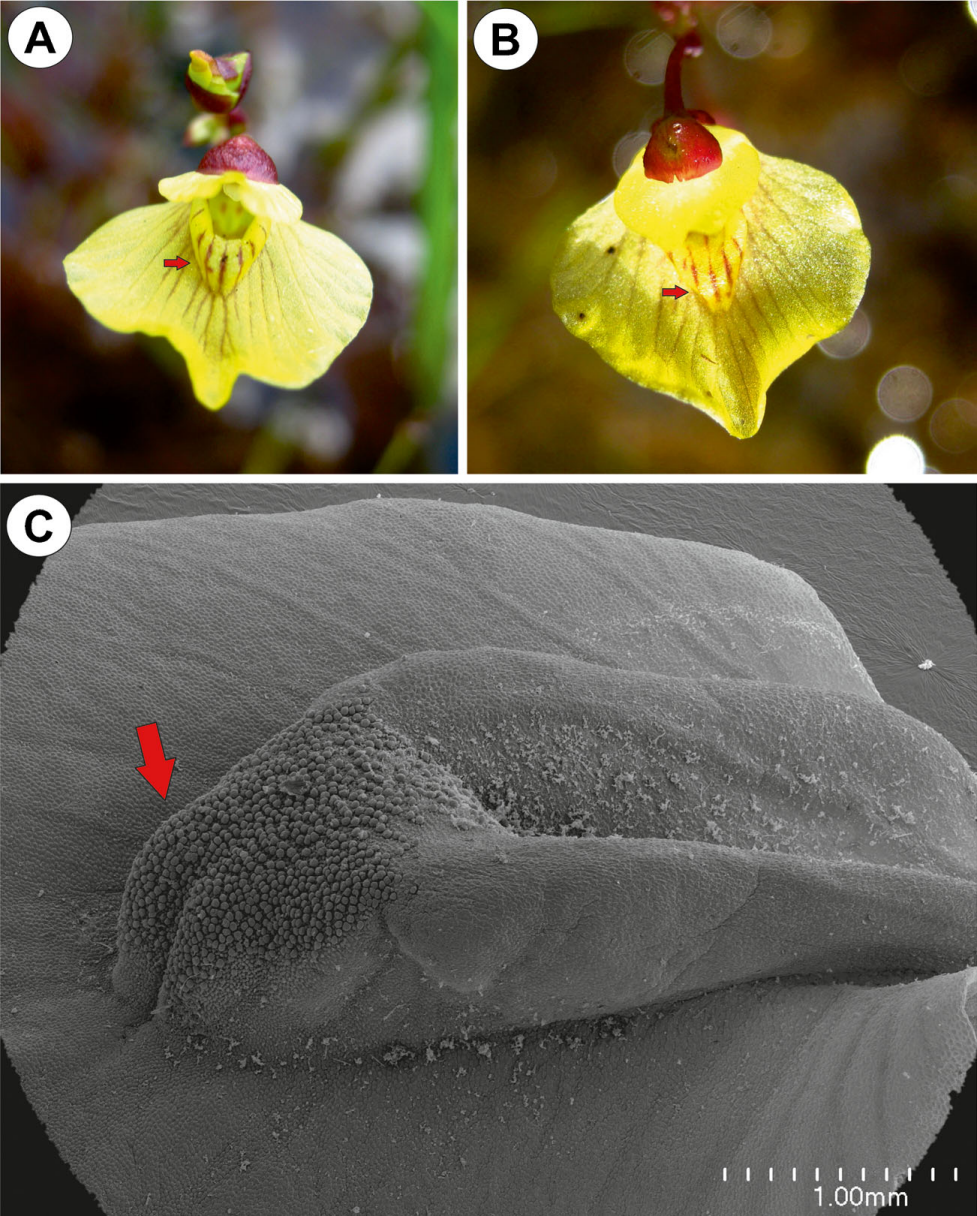
Floral morphology of *Utricularia bremii*. **a**–**b** Floral morphology of *Utricularia bremii* from the sand-pit Cep I in Suchdol nad Lužnicí–palate (*arrows*) with distinct nectar guides. **c** Morphology of the lower corolla lip–palate (*arrow*). *bar* = 1 mm

**Fig. 2:**
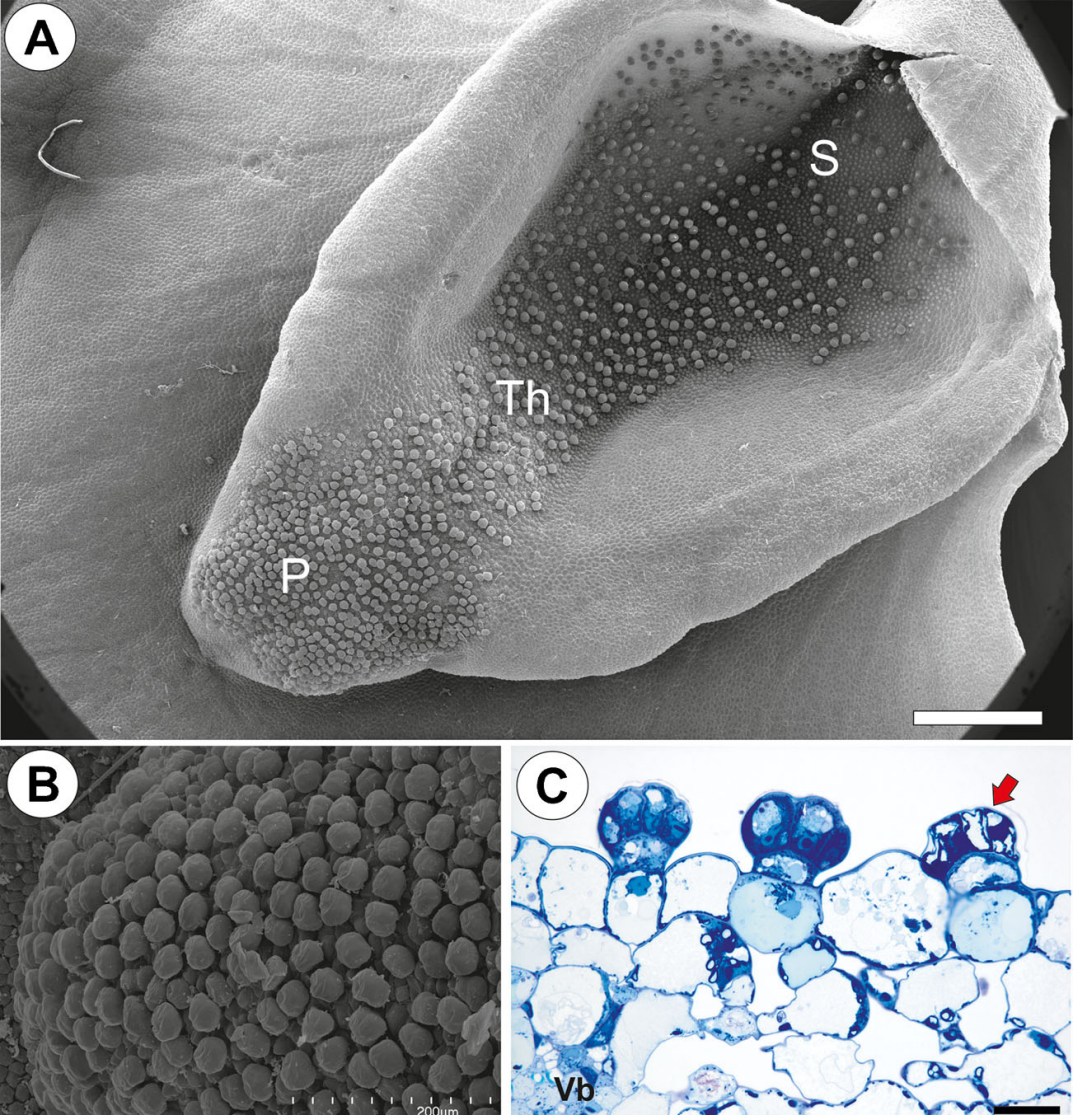
Micro-morphology of the lower corolla lip of a *Utricularia bremii* flower. **a** General morphology of the lower corolla lip–palate (*P*), throat (*Th*), spur (*S*); *bar* = 500 μm. **b** Palate with numerous glandular trichomes. *bar* = 200 μm. **c** Part of the section through the palate with glandular trichomes and subepidermal parenchyma showing large intercellular spaces; note that in one trichome, the head cells were degenerated (*arrow*), vascular bundle (*Vb*); *bar* = 20 μm

The corolla of *Utricularia bremii* was yellow in colour with red nectar marks on the palate. The lower corolla lip formed a wide platform. Its basal part formed palate (Fig.1a–c). The corolla palate was elongated and formed a rim of tissue, and its distal surface was covered with glandular trichomes (Figs. 1c and 2a–c). Glandular trichomes also occurred in the throat and the spur (Fig. 2a). No droplets of secretion were seen on the palate in the live material (Fig. 1a).

**Fig. 3:**
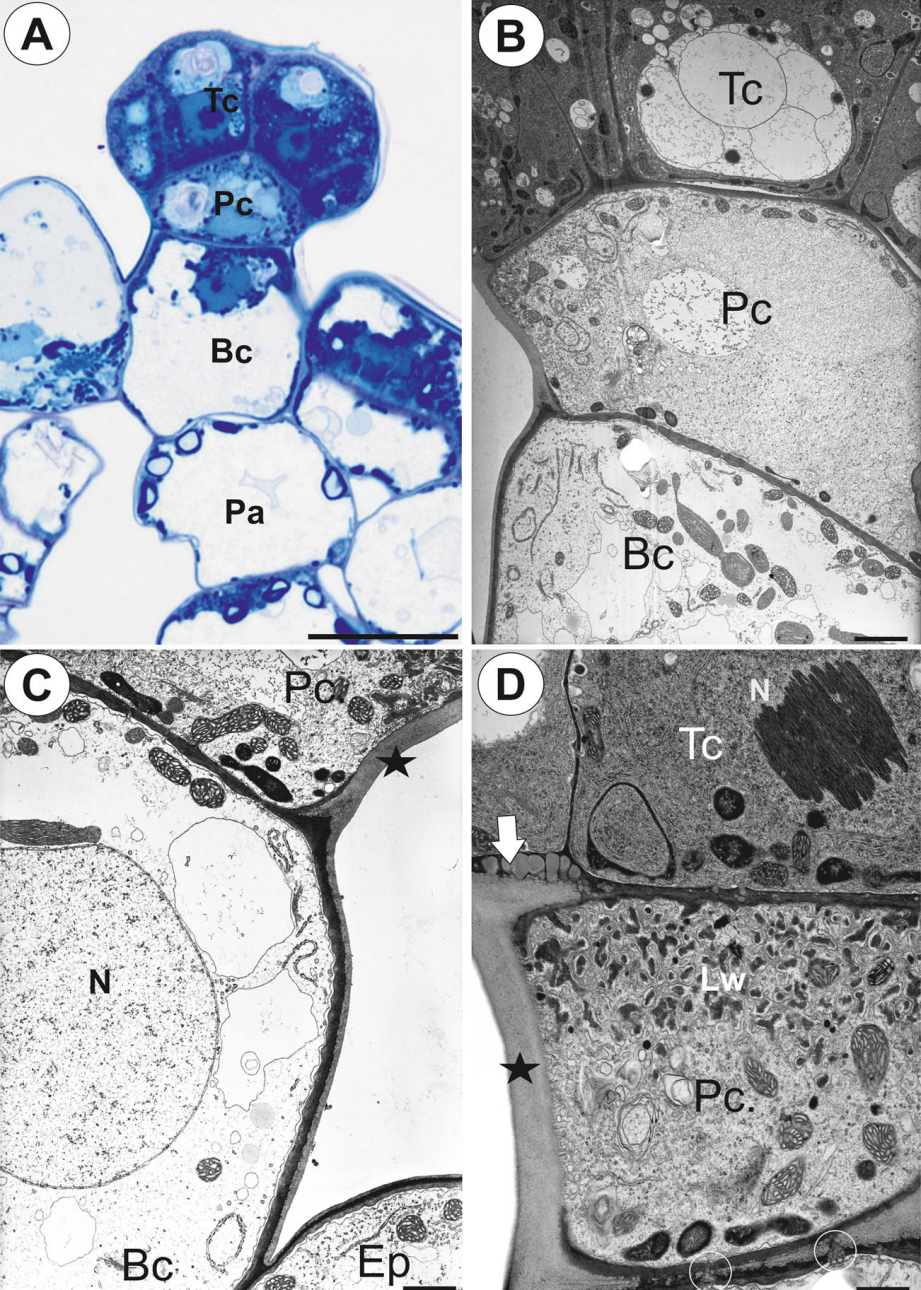
Structure of the palate trichomes of *Utricularia bremii*. **a** General structure of the glandular trichome; note that the head cells of the trichomes stain intensely with MB/AII–terminal = head cells (*Tc*), pedestal cell (*Pc*), basal cell (*Bc*), parenchyma cell (*Pc*) with starch grains; *bar* = 20 μm. **b** Longitudinal section showing terminal = head cells (*Tc*), pedestal cell (*Pc*), basal cell (*Bc*); *bar* = 2.80 μm. **c** Ultrastructure of a basal cell (*Bc*) and a pedestal cell (*Pc*)–nucleus of a basal cell (*N*), thickened impregnated anticlinal wall of a pedestal cell (*star*), epidermal cell (*Ep*); *bar* = 2.05 μm. **d** Section through a terminal cell (*Tc*) and a pedestal cell (*Pc*) showing wall ingrowths forming the labyrinth wall in a pedestal cell (*Lw*), thickened impregnated anticlinal wall of a pedestal cell (*star*), plasmodesmata between a pedestal and a basal cell (*circle*), cuticular deposits in the wall between a pedestal cell and a terminal cell (*arrow*), nucleus of a terminal cell (*N*); *bar* = 1.35 μm

Palate glandular trichomes were composed of a single basal cell, a unicellular stalk (pedestal cell) and a multi-celled head (Fig. 3a, b). All cell types were different in the case of vacuolisation and the degree of cytoplasm density (Fig. 3b). The basal cell was highly vacuolated, while the nucleus and most of the cytoplasm with the usual organelles were located in the upper part of the cell near the pedestal cell (Fig. 3a, c). The lateral wall of the basal cell lay partly embedded in the epidermis. Its outer part had a well-developed cuticle that was continuous with the cuticle of the epidermal cells and the cuticular deposits of the pedestal cell (Fig. 3 c, d). The basal cell also bordered the parenchyma cells, which contained starch grains (Fig. 3a).

**Fig. 4:**
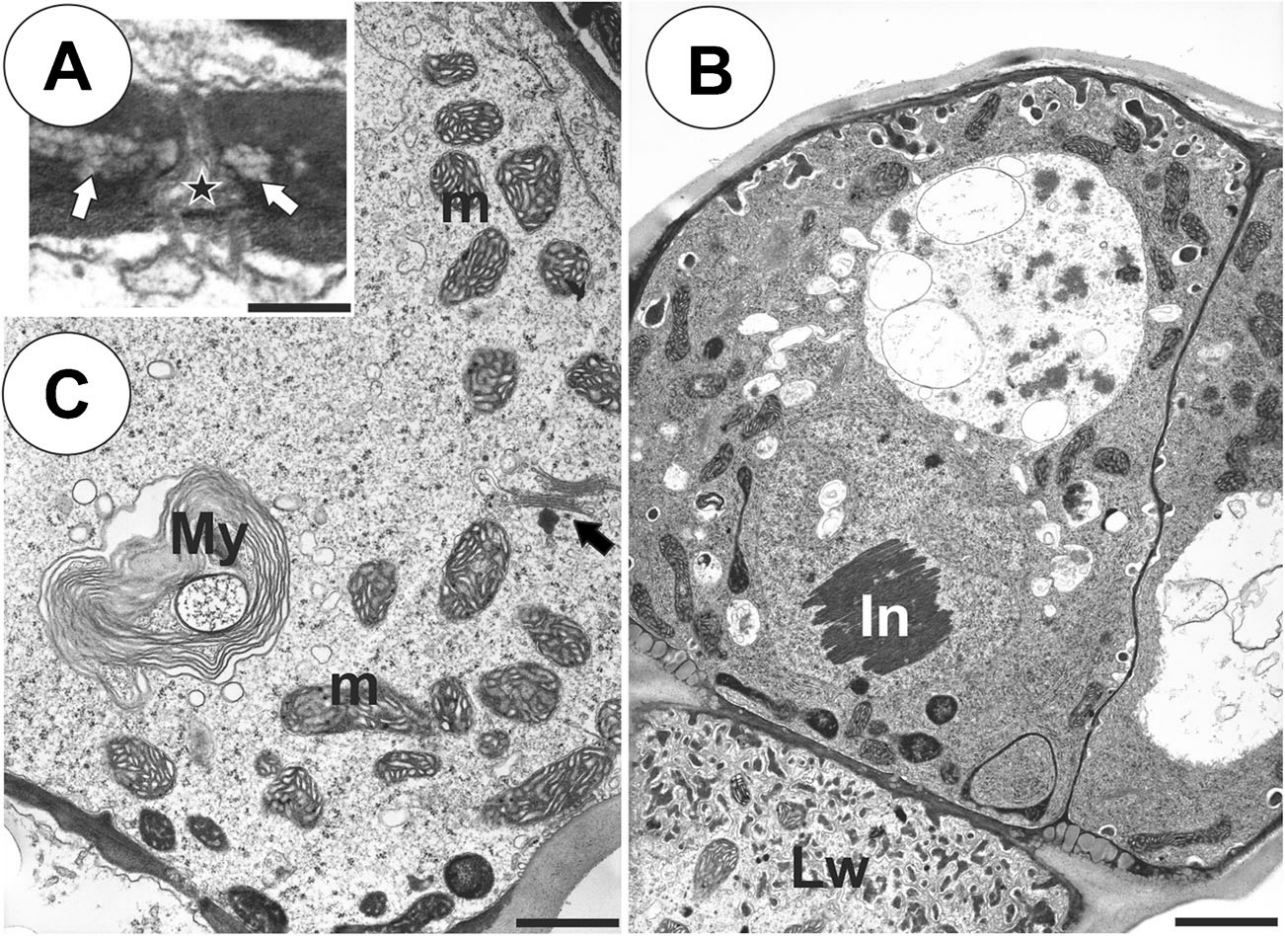
Ultrastructure of a glandular trichome from the palate of *Utricularia bremii*. **a** Branched plasmodesma (*star*) in the transverse walls between a basal cell and a pedestal cell, note the cuticular deposits (*arrows*); *bar* = 0.25 μm. **b** Ultrastructure of pedestal and terminal cells; note the well-developed labyrinth wall (*Lw*) in the pedestal cell, paracrystalline protein inclusion (*In*) in the nucleus of a terminal cell; *bar* = 1.35 μm. **c** Ultrastructure of a pedestal cell; note the numerous mitochondria (*m*), myelin-like figures (*My*) and dictyosomes (*arrow*); *bar* = 0.8 μm

The pedestal cell had a thick radial wall, which was impregnated with cutin (Fig. 3d). Cutin deposits also occurred in the transverse walls between the basal cell and the pedestal cell (Figs. 3d and 4a) and the pedestal cell and the terminal cells (Fig. 3d). Branched plasmodesmata occurred in the transverse walls between the basal cell and the pedestal cell (Fig. 4a). The plasmalemma in the region of the pedestal cell lateral wall displayed a wavy profile. In the pedestal cell, mitochondria had well-developed cristae (Fig. 4b), and they were associated with the cell wall ingrowths. A wall labyrinth (reticulate cell wall ingrowths) occurred on the transverse wall and partially on the lateral wall (Figs. 3d and 4c). Some myelin-like figures were also observed (Fig. 4b).

**Fig. 5:**
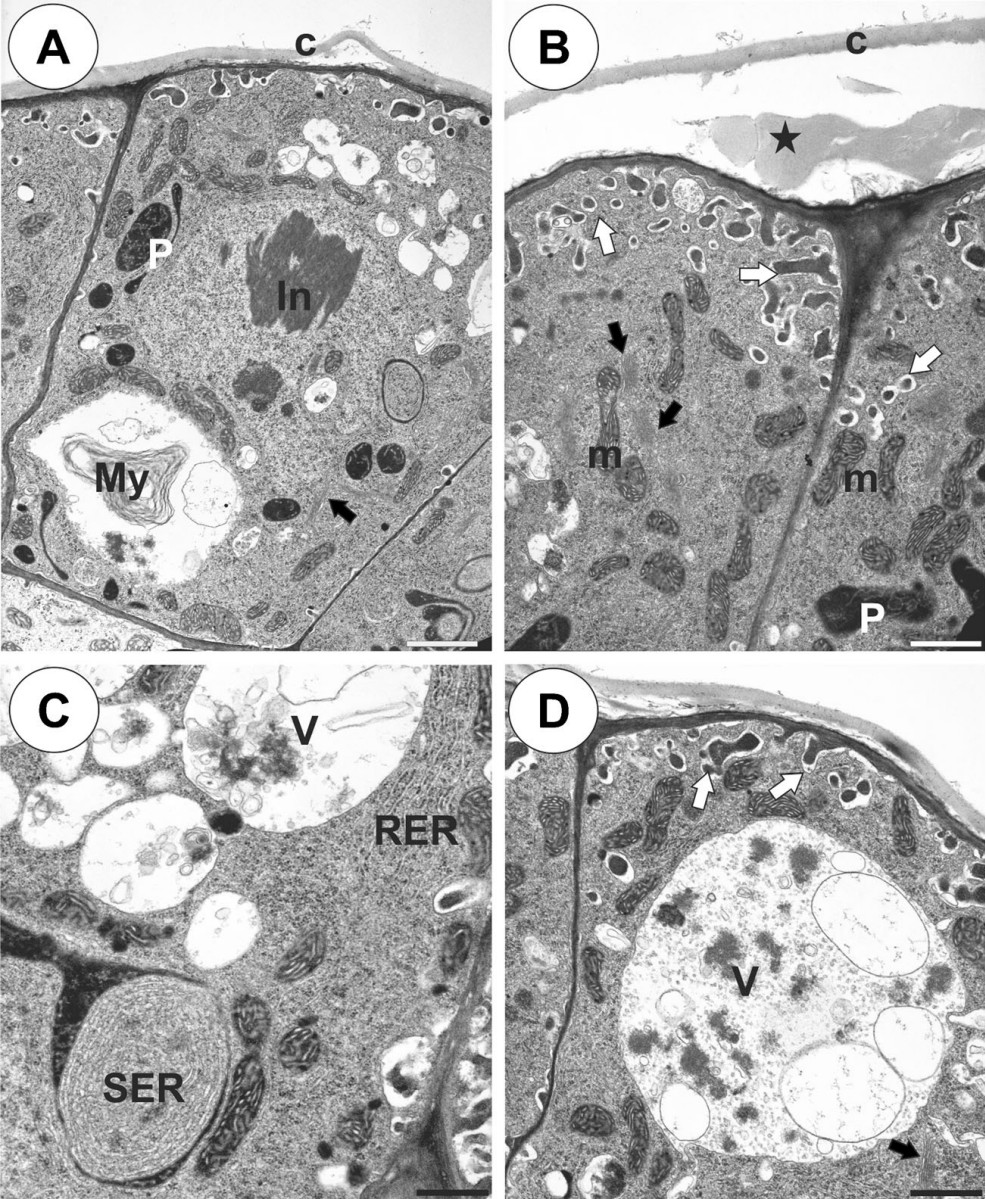
Ultrastructure of the glandular trichomes from the palate of *Utricularia bremii*. **a**–**d** Ultrastructure of terminal cells; note the paracrystalline protein inclusion (*In*) in the nucleus, dense cytoplasm with numerous plastids (*P*), mitochondria (*m*), dictyosomes (*black arrows*). In the vacuoles (*V*), there are myelin-like figures (*My*) and flocculent electron-dense material. Cell wall ingrowths (*white arrows*) on the inner surface of the outer wall but also on the inner walls between the terminal cells–smooth endoplasmic reticulum (*SER*), Rough endoplasmic reticulum (*RER*), cuticle (*c*), material in subcuticular space (*star*); **a** *bar* = 1.3 μm, **b** *bar* = 0.95 μm, **c** *bar* =0.55 μm, **d** *bar* = 0.80 μm

The protoplasts of the head cells were electron-dense and had a prominent, nucleus that contained a paracrystalline protein inclusion (Figs. 4c and 5a). Cell wall ingrowths not only occurred on the inner surface of the outer wall but also on the inner walls between the terminal cells (Figs. 4c and 5a–d). Intravacuolar myelin-like figures and flocculent electron-dense material were present (Figs. 4c and 5a, c–d). Mitochondria with well-developed cristae, profiles of rough endoplasmic reticulum (RER) and dictyosomes were common in the cytoplasm (Figs. 4c and 5a, c, d). Plastids were common and contained an electron-dense stroma; sometimes they were cube-shaped and were associated with the smooth endoplasmic reticulum (SER) (Fig. 5c). Part of the plastid contained small lipid globules (not shown). The thick cuticle frequently became distended and separated from the cell walls of the head cells, especially in the apical part of the cells (Fig. 5a, b). Some lipid or cutin material was observed between the cuticle and the cell wall (Fig. 5b). Although the protoplasts of the terminal cells collapsed in some trichomes (Fig. 2c), the protoplasts of the terminal cells were active in the neighbouring trichomes.

The cytoplasm of the head cells of the glandular trichomes stained deeply with methylene blue/azure II (Fig. 2c). Small lipid droplets were visible in the cytoplasm of the head cells in LM, but they were not frequent (not shown). SBB stained the cuticle of the terminal cells.

**Fig. 6:**
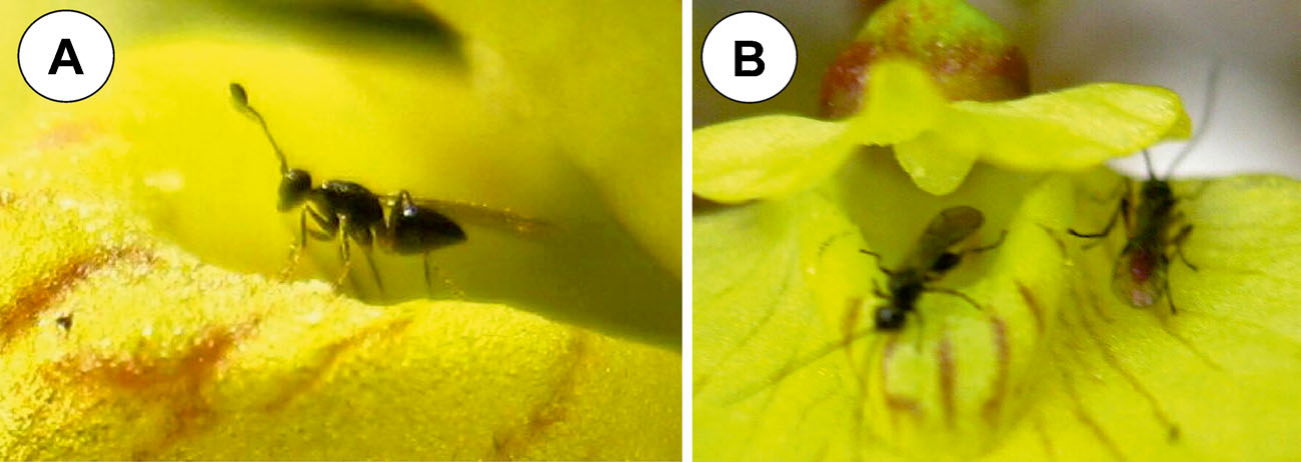
Visitors on the surface of the palate of *Utricularia bremii*. **a** Female, member of family Mymaridae, probably genus *Polynema* visiting *U. bremii* flower. **b** Members of family Braconidae visiting *U. bremii* flower

On the surface of the flowers of *Utricularia bremii*, visitors (small Hymenoptera: member of family Mymaridae, probably genus *Polynema* and members of family Braconidae) were observed (Fig. 6a, b). They penetrated the palate and the throat of the flowers. The flowers were of the open type (Fig. 6a, b).

### *Utricularia minor*

**Fig. 7:**
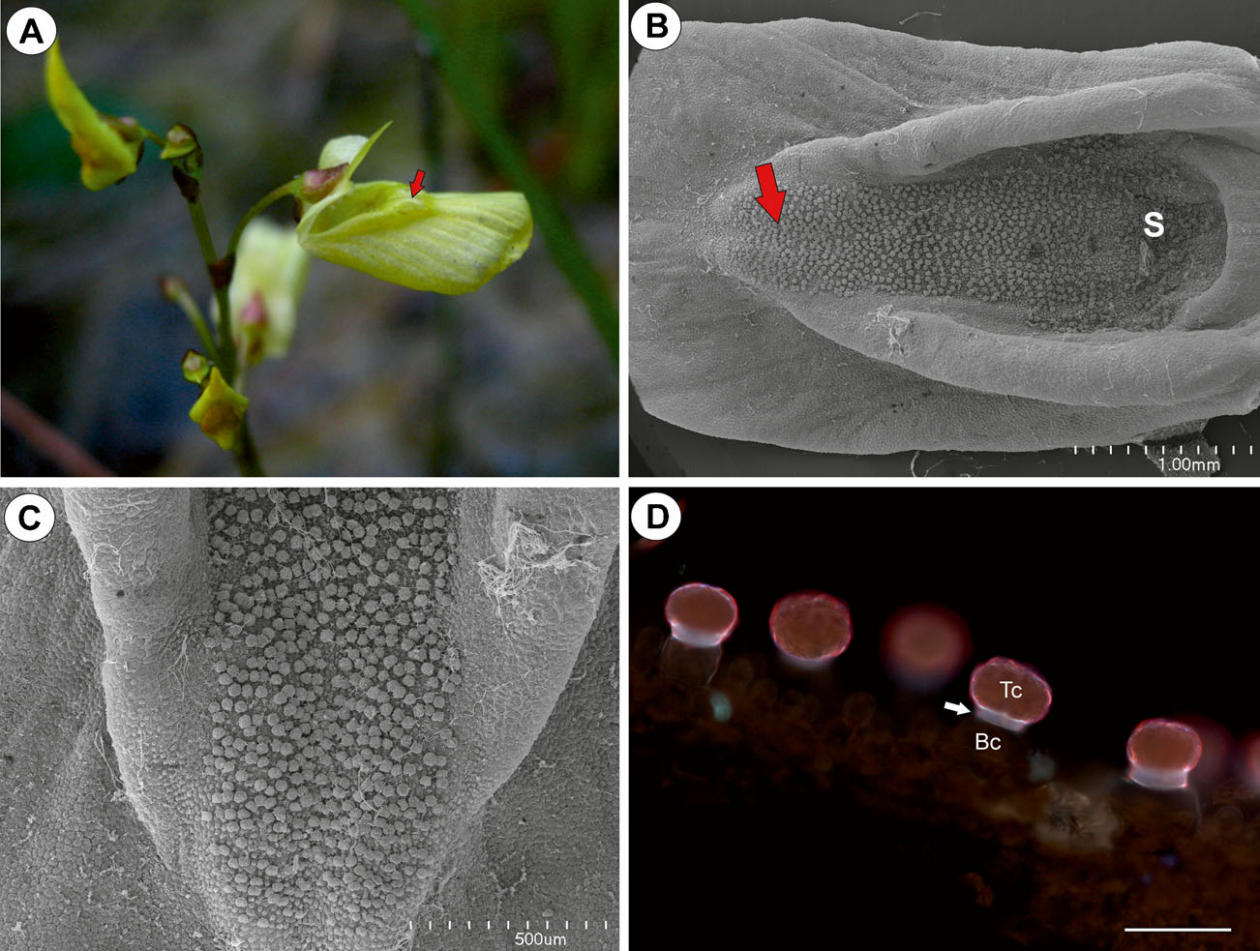
Floral morphology and structure of *Utricularia minor*. **a** General floral morphology of *Utricularia minor*–palate (*arrows*). **b** General morphology of the lower corolla lip–palate (*arrow*), spur (*S*); *bar* = 1 mm. **c** Palate with numerous glandular trichomes; *bar* = 500 μm. **d** Part of a section through the palate with glandular trichomes–terminal = head cells (*Tc*), pedestal cell (*arrow*), basal cell (*Bc*); *bar* = 50 μm

The flowers were of the open type (Fig. 7a). The corolla palate was elongated and formed a rim of tissue, and its distal surface was covered with glandular trichomes (Fig. 7b, c). The structure (Fig. 7d) and histochemistry of these trichomes resembled the glandular trichomes from the *U. bremii* palate. The palate parenchyma cells were rich in starch grains.

## Discussion

The described glandular trichomes of the palates of *U. bremii* and *U. minor* have an architecture that is similar to the glands of the unguentarium of *U. dunlopii* and the palates of *U. uniflora* and *U. paulinae* (Płachno et al. 2016) but are different from the long-stalked glandular trichomes of the palates of *U. cornigera* and *U. nelumbifolia* (Płachno et al. 2017). *U. bremii* and *U. minor* palates do not have the long papillae that are characters of the palates of *U. dichotoma*, *U. uniflora*, *U. paulinae* (Płachno et al. 2016), *U. cornigera* and *U. nelumbifolia* (Płachno et al. 2017). The characters of the ultrastructure glandular trichomes of the *U. bremii* palate are very similar to the trichomes of the unguentarium of *U. dunlopii*, and this might suggest that they have a similar function. Moreover, no nectar was observed on these trichomes in either of the species. Nonetheless, using TEM, we found lipid globules in the plastids of the terminal cells of *U. bremii*, similar to those that were found in *U. dunlopii*. The close association of the plastids with SER, which we observed in *U. bremii*, is a common character of the cells that produce terpenoids (Lange and Turner 2013). The tissue of the *U. dunlopii* unguentarium was rich in starch grains, similar to the starch that was recorded in the parenchyma cells of *U. bremii* palate. It is well known that starch is exploited as a source of energy in scent production (Vogel 1990; Nepi 2007) and that plastids that have starch grains are a common character of osmophore cells (e.g. Stern et al. 1987; Curry et al. 1991; Pansarin et al. 2009; Melo et al. 2010; Płachno et al. 2010; Antoń et al. 2012; Stpiczyńska and Davies 2016).

The occurrence of cell wall ingrowths in the head cells may be evidenced that the secreted material is transported via an eccrinous mode of secretion (Lüttge 1971), especially since we did not find neither the active dictyosomes nor the secretory vesicles. The occurrence of well-developed cell wall ingrowths in the pedestal cell indicates that there is intensive short-distance transport between the pedestal cell and the head cells (Gunning and Pate 1969).

Although the cell wall ingrowths are a ubiquitous feature of the nectary cells (e.g. Schnepf and Pross 1976; Kronestedt and Robards 1987; Stpiczyńska et al. 2012), these structures are rarely recorded in the osmophore cells possibly because a granulocrine mode of secretion is most often suspected in the osmophores (Caissard et al. 2004 and literature therein). However, recently Kowalkowska et al. (2016) found cell wall ingrowths in cells in the petals of *Bulbophyllum weberi* Ames, which may function as osmophores.

In *Utricularia* cell wall ingrowths, a terminal cell and a pedestal cell with various types of secretory trichomes that are connected with carnivory (on the external and internal trap surface) and also sessile trichomes on the stolon and phylloclade surface, which play role in nutrients absorption from water, were recorded (e.g. Fineran and Lee, 1975, 1980; Fineran 1980, 1985; Płachno and Jankun, 2004). Thus, cell wall ingrowths are common character of secretory or absorptive trichomes in the *Utricularia*.

In both trichomes on the unguentaria of the *U. dunlopii* and *U. bremii* palates, TEM observations revealed that the cuticle frequently became distended and separated from the cell walls of the head cells. Thus, a subcuticular space is formed, which suggests a subcuticular accumulation of secretions.

Intravacuolar myelin-like figures were recorded in variety of plant tissues (Davies et al. 1992, and references therein), also is secretory cells e.g. in orchid elaiophore cells (Davies and Stpiczyńska 2009). These structures are probably related to tissue differentiation and senescence (Davies et al. 1992). However, other authors suggested that myelin-like figures might be an artefact caused by tissue fixation for TEM (e.g. Hwang and Chen 1997).

In contrast to the large flowered species that were analysed, *U. reniformis* (Clivati et al. 2014), *U. cornigera* and *U. nelumbifolia* (Płachno et al. 2017), which need large, strong pollinators, both *U. bremii* and *U. minor* are of an open flower type. Thus, *U. bremii* and *U. minor* flowers can be penetrated by small, weak insects, which then easily have access to their generative structure. In the case of *U. bremii*, the observed insects cannot be called pollinators because this species is considered to be sterile as a consequence of the disturbations that occurred during their microsporogenesis (Casper and Manitz, 1975). Recently, Beretta and co-authors (2014) showed that over 95% of the pollen grains in *U. bremii* are malformed and anomalous. This species reproduces vegetatively via shoot fragmentation and turions (Taylor 1989).

## Conclusion

Only one type of glandular trichome occurred on the palates of *U. bremii* and *U. minor*, and this is different than the *U. cornigera* and *U. nelumbifolia* palates, which have various glandular trichomes and well-developed papillae. Due to the similarity of the glandular trichomes of the *U. bremii* and *U. minor* palates to the *U. dunlopii* unguentarium trichomes, we suggest that the *U. bremii* and *U. minor* palates may also act as unguentaria. Given the enormity of the *Uricularia* genus (about 240 species on six continents), we realise that a much greater concerted effort to characterise the anatomical, histochemical and ultrastructural characteristics of the floral secretory tissue is required before arriving at any generalisations. Thus, we treat our study as one more step toward developing an understanding of both the palate function and pollination syndrome in the *Utricularia* genus.
